# Clinical Study on the Etiology of Postthyroidectomy Skin Sinus Formation

**DOI:** 10.1155/2017/5283792

**Published:** 2017-03-12

**Authors:** Shan Jin, Wuyuntu Bao, Oyungerel Borkhuu, Yun-Tian Yang

**Affiliations:** Department of General Surgery, Affiliated Hospital of Inner Mongolia Medical University, Tongdao North Rd 1, Hohhot 010050, Inner Mongolia Autonomous Region, China

## Abstract

*Background*. Thyroidectomy is one of the most frequently performed surgical procedures worldwide. Despite technical advances and high experience of thyroidectomy of specialized centers, it is still burdened by a significant rate of postoperative complications. Among them, the skin sinus formation is an extremely rare postthyroidectomy complication. Here, we first report the incidence of the skin sinus formation after thyroidectomy to identify the causes for skin sinus formation after thyroidectomy and to discuss its prevention and treatment options.* Methods*. A retrospective analysis was carried out of patients who underwent excision operation of fistula for postthyroidectomy skin sinus formation. Data were retrieved from medical records department of the Affiliated Hospital of Inner Mongolia Medical University.* Results*. Of the 5,686 patients who underwent thyroid surgery, only 5 patients (0.088%) had developed skin sinus formation. All 5 patients successfully underwent complete excision of fistula.* Conclusion*. Infection, foreign body, thyroid surgery procedure, combined disease, and iatrogenic factors may be related with skin sinus formation after thyroidectomy. To reduce the recurrence of postoperative infections and sinus formation, intra- and postoperative compliance with aseptic processing, intraoperative use absorbable surgical suture/ligature, repeated irrigation and drainage, and postoperative administration of anti-inflammatory treatment are to be followed.

## 1. Background

Thyroidectomy is one of the most frequently performed surgical procedures worldwide. The overall incidence of postthyroidectomy complications ranges from 6% to 28% [1–4], and the mortality rate ranges from 0% to 1.92% [5–9]. Common postthyroidectomy complications include postoperative bleeding, recurrent laryngeal nerve injury, superior laryngeal nerve injury, hypoparathyroidism, chylous fistula, surgical site infection, and formation of keloid and skin sinus. Among them, skin sinus formation is an extremely rare postthyroidectomy complication. Here, we first report the incidence of the skin sinus formation after thyroidectomy to identify the causes for skin sinus formation after thyroidectomy and to discuss its prevention and treatment options.

## 2. Methods

### 2.1. Ethics Statement

This study was approved by the ethics committee of Inner Mongolia Medical University (no. YKD2014063). Two patients gave written informed consent to publish their case clinical photograph.

### 2.2. Subjects

Patients who experienced skin sinus formation after they underwent thyroidectomy at Affiliated Hospital of Inner Mongolia Medical University, Inner Mongolia Autonomous Region, Hohhot, China (between March 2009 and March 2013 were included in the study). Data were retrieved from the hospital's medical records department as well as the surgical theater procedure register. The data were retrospectively analyzed and reviewed.

### 2.3. Diagnosis and Patient Characteristics

All patients underwent thyroid surgery. When surgical stitches were removed, some patients experienced surgery incision discomfort or even pain, neck skin ulceration, and pus. Although their wounds were healing and infections were controlled by frequent dressing, ulceration and pus reappeared. The infected site repeatedly had scars and sinus formation, and some patients had general malaise and fever symptoms. On physical examination, cervical surgery incision scar and external fistula were seen. Size of the external fistula, circumferential skin, and shape of the effluent were observed along with the effluent for cytology bacteriology. Soft tissue around the fistula was touched and observed to determine masses, scars, and cords. Laboratory tests were performed, especially to observe the trend of white blood cell count, which increased in some patients. Ultrasound and computer tomography (CT) examinations were performed to determine sinus shape, out of shape, length, and location of the sinus termination end. In addition, oral and laryngopharyngeal cavities were examined to exclude other possibilities of fistula.

### 2.4. Management

During the acute phase of inflammation, patients were treated with frequent dressings, adequate drainage, and secretion for bacterial culture. If necessary, anti-inflammatory treatment could be administrated. Complete excision of fistula was performed until the inflammation subsided, and the resected specimens were sent for pathological examination to confirm the diagnosis and exclude the presence of cancer.

## 3. Results and Discussion

### 3.1. Baseline and Demographic Characteristics

Of the 5,686 patients who underwent thyroid surgery, only 5 patients (0.088%) had developed skin sinus formation. All the patients were female, and their median age was 54.4 (range: 44~70) years. Primary thyroid disease was nodular goiter in 3 patients (60%) and Hashimoto's disease in 1 patient (20%). Nodular goiter was associated with Hashimoto's disease in 1 patient (20%), but no patients had a thyroid cancer. The mean time from thyroid surgery to pus rupture was 16.2 (range: 11 to 21) days, and the mean disease course was 2.6 (range: 1 to 4.5) years. Two patients (40%) had diabetes. Bacterial culture test of sinus secretions showed that 3 patients (60%) were associated with* Staphylococcus aureus* (SA) infection and 1 patient (20%) was associated with anaerobic infections (Table 1).

### 3.2. Surgery Related Status and Result of the Management

**Figure 1:**
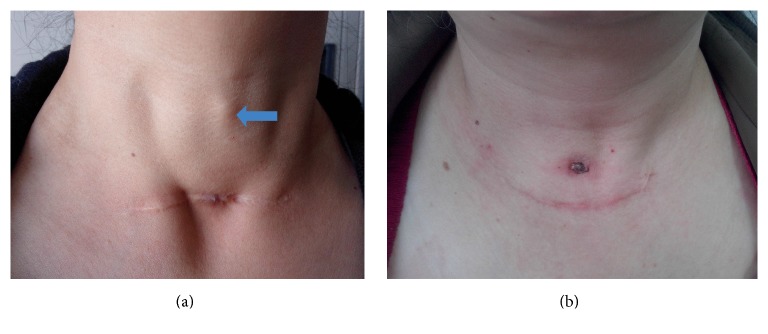
Clinical photograph of postthyroidectomy skin sinus formation. (a) Sinus was located in primary thyroid surgery incision line; (b) sinus was located above the primary thyroid surgery incision line; arrow: abscess under the skin.

Patients' primary surgical procedures included partial thyroidectomy and near total thyroidectomy. During the acute phase of inflammation, patients' condition was treated with frequent dressings, adequate drainage, anti-inflammatory drugs, and symptomatic treatment. Complete excision of fistula was performed until the inflammation subsided. Sinus was located in primary thyroid surgery incision line in 3 patients (60%), above the primary thyroid surgery incision line in 1 patient (20%), and in the drainage outlet in 1 patient (20%) (Figure 1). Mean size of sinus was 0.68 × 0.68 cm (range: 0.5 × 0.5 cm to 1 × 1 cm) in entrance diameter, and the sinus tract extended to an average of 5.5 cm (range: 4 cm to 7.5 cm). Sinus termination end was located at residual thyroid gland in all 5 patients and the foreign body–sutures reaction was found in all 5 patients (100%) (Table 2).

**Figure 2:**
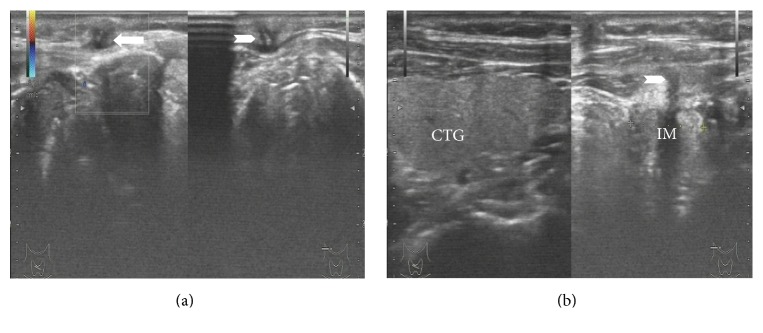
Transverse US image shows sinus formation. CTG: contralateral thyroid gland; IM: inflammatory mass; arrow: abscess under the skin; arrowheads: sinus.

**Figure 3:**
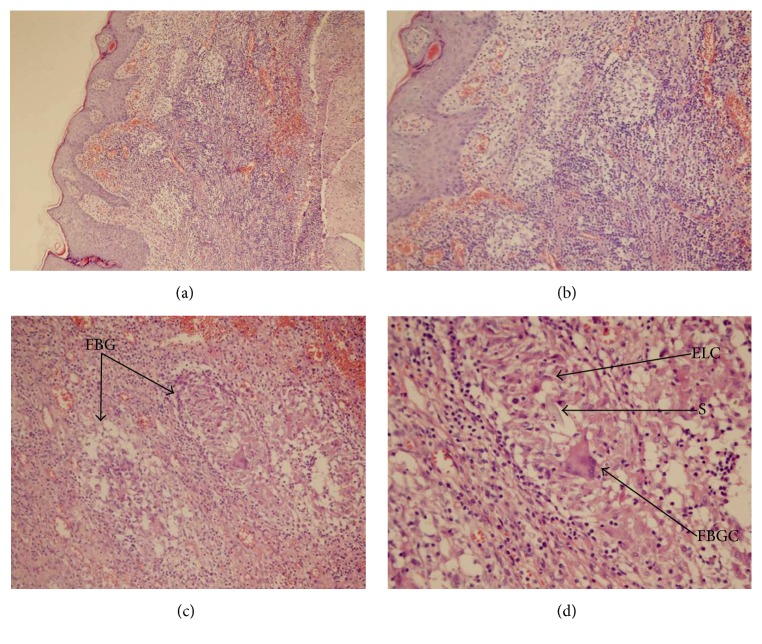
Photomicrograph. (a) Sinus parietal pathology HE ×100; (b) sinus parietal pathology HE ×200; (c) foreign body granuloma formation HE ×200; (d) foreign body granuloma formation HE ×400; FBG: foreign body granuloma; ELC: epithelial-like cell; S: suture; FBGC: foreign body giant cell.

During the operation, sinus tract crossing fusiform incision was performed, and the cords of scar tissue were attentively touched. If necessary, the probe or external fistula was injected with methylene blue injection to determine the sinus shape and length, combined with preoperative ultrasound and CT examinations (Figure 2). Complete excision of fistula was performed. Simultaneously, thyroid infected lesion, granulation tissue, necrotic tissue, and foreign body were removed. The occurrence of misdiagnosis was prevented by sending the resected specimens for pathological examination (Figure 3). All 5 patients (100%) had complete excision of fistula. During the operation, the incision was repeatedly irrigated. Absorbable suture, ligation, and drainage were performed; and postoperative administration of anti-inflammatory treatment was provided. All patients were discharged without complications. Mean postoperative duration time was 20 (2 to 35) months with no recurrences.

## 4. Discussion

Skin sinus formation is an extremely rare postthyroidectomy complication. In 1949, Donato first reported one case of skin sinus formation after thyroidectomy [10]. In 1986, Vesely et al. reported that the formation of a sinus tract in the neck after a subtotal thyroidectomy appeared to be a much less common complication. However, the incidence of sinus tract formation after thyroid surgery is unknown at present [11]. After Okumuş and Bilgin-Karabulut reported a large sinus on the neck in 1 patient as a rare complication after thyroidectomy [12]. In this study, only 5 patients had skin sinus formation among the 5,686 patients (0.088%) who underwent thyroidectomy during the study period. This explains that the formation of a sinus tract in the neck after thyroidectomy appears to be a much less common complication than the previously reported complications such as postoperative bleeding, recurrent laryngeal nerve injury, superior laryngeal nerve injury, hypoparathyroidism, chylous fistula, and surgical site infection. The following factors may be related with skin sinus formation after thyroidectomy: infection, foreign body, thyroid surgery procedure, combined disease, and iatrogenic factors. The incidence of surgical site infection after thyroidectomy ranges from 0.3% to 3.2% per the available literature [6, 13, 14]. Thyroid gland and circumferential interspace infection are possible causes of skin sinus formation after thyroidectomy. Infectious diseases of the thyroid gland are uncommon because of factors which resist infection such as well-developed capsule, high iodine content of the gland, and prosperous lymphatic and vascular supply [15, 16]. However, thyroidectomy could damage the gland anatomy, and it may weaken the protective effect of the above factors. In this study, 5 patients (100%) had recurrent postoperative abscess formation and rupture, 3 patients (60%) had pus culture bacterial infections, and 1 (20%) had anaerobic infections. These indicate the involvement of different types of bacteria symbiosis and reproduction in sinus formation. This may be one of the reasons why the infection is persistent and wound healing is not proper. Vesely et al. reported that this complication could be caused by a foreign body reaction to the sutures [11]. In this study, the foreign body–sutures reaction was found in all 5 patients (100%). This explains that sutures may become the attachment for the colonized bacteria or conducive to the formation of bacterial biofilms and become a source of secondary infection. It may be the main reasons for repeated occurrence of abscess. In this study, primary surgical procedures were partial or subtotal resection of the thyroid in all 5 patients (100%); and terminal end of sinus formation was located on the residual thyroid, which indicated that residual thyroid lesions could become the focus of infection. Total thyroidectomy may reduce the formation of skin sinus effectively. Two patients (40%) had comorbid diabetes mellitus. Diabetes affects the body immunity and reduces the body's resistance to bacteria and defense. Patients with diabetes are easy to be infected and difficult to control infection. Iatrogenic factors are also the main reasons of skin sinus formation after thyroidectomy. Thyroidectomy is a clean neck surgery, and the risk of surgical site infection depends mainly on the quality of pre- and postoperative care and use of sterile technique [13]. Preoperative antibiotics do not affect the incidence of surgical site infection [13]. Hence, it is important to improve sterile technique and sufficient surgical site rinsing. Moreover, the use of nonabsorbable suture may increase incidence of the sinus formation. In this study, terminal end of sinus tract was at superior thyroid gland in 3 patients (60%) and in the middle of the thyroid gland in 2 patients (40%), which is maybe indicating more superior thyroid vascular bleeding. In order to reduce intraoperative bleeding, surgeons could use nonabsorbable suture to suture gland/ligate vessels. Intraoperative use of nonabsorbable sutures may increase the incidence of postoperative surgical site infection and skin sinus formation. Use of hemostatic materials during the operation may not increase the incidence of postoperative surgical site infection and neck skin sinus formation. Steroids, smoking, cancer, and chemoradiotherapy may increase fistula incidence after surgical treatment [12, 17]. Reducing skin sinus formation after thyroidectomy may be related with more accurate knowledge of the thyroid anatomy and skilled surgical handling and sterile technique. Treatment methods of common sinus tracts include chemocauterization [18], sclerotherapy [19], negative pressure wound therapy [20], and complete excision of the fistula. For the patients with cervical sinus formation after the thyroid surgery, adequate drainage, anti-inflammatory, and symptomatic treatment can be used during the acute phase of inflammation frequent dressings; but they should not undergo surgery. Complete excision of fistula should be performed until the inflammation subsides. The principles of surgical treatment involve elimination of cause, removal of foreign body, resecting the gland foci, and complete excision of sinus. Skin sinus infection after thyroidectomy is different from other types of skin sinus infection. The infection travels along the path of least resistance which is guided by muscle attachments and distribution of loose connective tissue in that area. In this study, the mean length of the sinus was 5.5 cm (range: 4 cm to 7.5 cm) and the mean outer size of fistula was 0.68 × 0.68 cm (range: 0.5 × 0.5 cm~1 × 1 cm), which was shorter than other types of skin sinus formation. The skin sinus after thyroidectomy is out of shape, less tortuous, and relatively easy to perform a surgery. For the recurrent infections and unclear anatomy, it is difficult to reveal the whole sinus. Methylene blue, microscope guidance, probe guidance, nelaton catheter, and Fogarty catheter can be used to help exposure [21–25]. In this study, 5 patients (100%) successfully underwent complete excision of fistula, and 2 patients (40%) simultaneously underwent ipsilateral gland resection. Five patients (100%) were discharged home without any further complications. Skin sinus formation is an extremely rare complication after thyroidectomy, but its incidence is on the rise. Abscesses and recurrent ulceration may cause severe pain to the patients. Thus it is more important to prevent its occurrence than treatment.

## 5. Conclusion

To reduce the recurrence of postoperative infections and sinus formation, intra- and postoperative compliance with aseptic processing, intraoperative use absorbable surgical suture/ligature, repeated irrigation and drainage, and postoperative administration of anti-inflammatory treatment are to be followed.

## Figures and Tables

**Table 1:** Clinical characteristics.

Patient	Age	Gender	Primary thyroid disease	Operation time (min)	Intraoperative bleeding (mL)	Thyroid surgery appears to rupture pus time	Secretion bacterial culture	Total duration	Combined disease
1	52	Female	NG	78	180	20 d	*Staphylococcusaureus*	4.5 y	Diabetes
2	56	Female	NG	55	90	15 d	—	3 y	Diabetes
3	70	Female	NG associated with HT	84	210	14 d	*Staphylococcusaureus*, streptococcus, anaerobes	2.5 y	Hypertension
4	50	Female	HT	50	80	21 d	—	2 y	—
5	44	Female	NG	52	110	11 d	*Staphylococcusaureus*	1 y	—

—: no bacterial culture or no combined disease; HT: Hashimoto's disease; NG: nodular goiter; y: year.

**Table 2:** Surgery related status.

Patients	Primary surgical procedure	Sinus location	Size of the sinus	Extension of sinus tract	Sinus termination end	Foreign body	Surgical procedure	Histopathological	Postoperative followup time
1	Both partial thyroidectomy	In primary incision line	0.5 × 0.5 cm	5 cm	SRT	Sutures	CEF	Compliance with sinus No cancer	20 months
2	Both partial thyroidectomy	In primary incision line	1 × 1 cm	6 cm	MRT	Sutures	CEF	Compliance with sinus No cancer	35 months
3	Both subtotal thyroidectomy	Drainage outlet	0.8 × 0.8 cm	7.5 cm	SLT	Sutures	CEF + right residual thyroidectomy	Compliance with sinus No cancer	24 months
4	Both subtotal thyroidectomy	above the incision line	0.6 × 0.6 cm	5 cm	SRT	Sutures	CEF	Compliance with sinus No cancer	2 months
5	Left subtotal thyroidectomy	In primary incision line	0.5 × 0.5 cm	4 cm	MLT	Sutures	CEF + left residual thyroidectomy	Compliance with sinus No cancer	19 months

CEF: complete excision of fistula; SLT: superior left thyroid; SRT: superior right thyroid; MLT: middle left thyroid; MRT: middle right thyroid.
